# Sleep architecture and serum biomarker heterogeneity in obstructive sleep apnea: a cross-sectional study

**DOI:** 10.1093/sleepadvances/zpag046

**Published:** 2026-05-22

**Authors:** Zafer Hasan Ali Sak, Mahmut Ülger, Serif Kurtulus, Hakim Celik, Hamza Erdogdu, Hamdiye Turan

**Affiliations:** Department of Pulmonary Medicine, Harran University Faculty of Medicine, Sanliurfa, Turkiye; Department of Pulmonary Medicine, Harran University Faculty of Medicine, Sanliurfa, Turkiye; Department of Pulmonary Medicine, Harran University Faculty of Medicine, Sanliurfa, Turkiye; Department of Physiology, Harran University Faculty of Medicine, Osmanbey Campus, Sanliurfa, Turkiye; Department of Biostatistics, Harran University Faculty of Medicine, Sanliurfa, Turkiye; Department of Pulmonary Medicine, Harran University Faculty of Medicine, Sanliurfa, Turkiye

**Keywords:** obstructive sleep apnea, sleep architecture, REM sleep, hypoxemia, neurodegeneration

## Abstract

**Study Objectives:**

To determine whether sleep architecture is associated with heterogeneity in serum biomarkers of neuronal injury, astroglial stress, tau-related pathology, and neuroplasticity in adults with obstructive sleep apnea (OSA).

**Methods:**

Adults referred for suspected OSA underwent overnight polysomnography and next-morning fasting serum sampling. Primary analyses were restricted to participants with OSA (apnea-hypopnea index [AHI] ≥5 events/h; *n* = 82). Serum neuron-specific enolase (NSE), neurofilament light chain (NfL), brain-derived neurotrophic factor (BDNF), cAMP response element-binding protein 1 (CREB1), S100 calcium-binding protein B (S100B), glial fibrillary acidic protein (GFAP), and (phosphorylated microtubule-associated protein tau) p-Tau/human pMAPT/pTAU were measured by enzyme-linked immunosorbent assay (ELISA). We first examined whole-group correlations between respiratory burden indices (AHI, ODI, and T90) and biomarkers, then fit age- and sex-adjusted linear models with continuous sleep-stage percentages. Median-split stage analyses and stage-stratified correlations were treated as exploratory sensitivity analyses.

**Results:**

Biomarker availability ranged from 70 to 71 participants. Lower rapid eye movement (REM) percentage was independently associated with higher ln(NSE) (beta = −0.0138 per 1 per cent REM; 95% CI = −0.026 to −0.001; *p* = .033). Whole-group BDNF correlated inversely with ODI (rho = −0.299, *p* = .011) and AHI (rho = −0.277, *p* = .020). In sensitivity analyses, REM-AHI was positively associated with p-Tau/human pMAPT/pTAU (beta = 1.799, *p* = .006) and GFAP (beta = 2.094, *p* = .024). Interaction terms testing formal effect modification by REM per cent or N3 per cent were directionally consistent with the hypothesis but did not reach conventional significance. After Bonferroni correction, only the short-REM AHI-BDNF correlation remained significant among exploratory stage-stratified correlations.

**Conclusions:**

Reduced REM sleep showed the clearest and most reproducible relationship with an adverse peripheral biomarker profile in OSA, specifically higher serum NSE. Sleep architecture may contribute to biomarker heterogeneity, but evidence for stage-dependent modification of hypoxemia-biomarker relationships remains exploratory.

**Statement of Significance**

Patients with obstructive sleep apnea who have similar apnea severity can still differ in biologically relevant sleep architecture. In this cohort, lower rapid eye movement (REM) sleep showed the most reproducible relationship with a peripheral biomarker profile suggestive of greater neuronal injury, reflected by higher serum neuron-specific enolase. Whole-group analyses also showed lower brain-derived neurotrophic factor with greater respiratory burden. Direct interaction tests suggested, but did not definitively confirm, that reduced REM or N3 sleep may strengthen adverse hypoxemia-biomarker relationships. These findings support sleep architecture as a source of biomarker heterogeneity in obstructive sleep apnea, but they should be interpreted as exploratory and hypothesis-generating pending longitudinal confirmation.

## Introduction

Obstructive sleep apnea (OSA) is characterized by recurrent partial or complete upper-airway obstruction during sleep, resulting in chronic intermittent hypoxia and sleep fragmentation [1, 2]. These stressors can promote oxidative stress, neuroinflammation, impaired synaptic plasticity, and cognitive dysfunction, particularly in memory-related domains [1–3]. Prospective evidence further suggests that sleep-disordered breathing with intermittent hypoxemia is associated with an increased risk of mild cognitive impairment and dementia [4].

We selected a biomarker panel intended to capture complementary aspects of neuronal injury (NSE, NfL), astroglial stress (S100B, GFAP), tau-related pathology (p-Tau/human pMAPT/pTAU), and neuroplastic/neurotrophic signaling (BDNF, CREB1). NSE is a glycolytic enzyme enriched in neurons and is commonly used as a peripheral marker of neuronal soma or cell-body injury. NfL reflects neuroaxonal injury, GFAP and S100B reflect astroglial stress, and pathological phosphorylation of MAPT (microtubule-associated protein tau) is linked to Alzheimer-type tauopathy [5–12].

BDNF is a neurotrophin involved in neuronal survival and synaptic plasticity. Circulating BDNF findings in OSA are heterogeneous, and both sleep architecture and continuous positive airway pressure treatment may influence peripheral levels [13–18]. CREB1 was included as an exploratory peripheral marker related to the BDNF/CREB neuroplasticity pathway, although circulating CREB measures are less established than tissue-based, cerebrospinal fluid, or intracellular phospho-CREB assessments [19].

Accordingly, we aimed to determine whether sleep-stage distribution is associated with heterogeneity in serum biomarker profiles in adults with OSA and whether stage composition may influence the relationship between nocturnal respiratory burden and these biomarkers.

## Materials and methods

This cross-sectional study is reported in accordance with the STROBE statement.

### Participants

Between 2021 and 2023, 91 adults (≥18 years) referred to the Harran University Sleep Clinic for suspected sleep-disordered breathing underwent overnight in-laboratory polysomnography and next-morning fasting blood sampling. Symptoms prompting referral included snoring, witnessed apnea, and excessive daytime sleepiness.

Exclusion criteria were a prior diagnosis of dementia or other neurological disorders, ongoing treatment for dementia, and a history of neuroendocrine tumors. Written informed consent was obtained from all participants. This study was approved by the Harran University Clinical Research Ethics Committee (decision date: February 7, 2022; approval date: March 22, 2022) and was conducted in accordance with the Declaration of Helsinki.

For primary analyses, the analytic OSA cohort was defined as participants with an apnea-hypopnea index (AHI)≥5 events/h (*n* = 82).

### Polysomnography protocol

All participants underwent overnight, video-monitored polysomnography using a 55-channel Alice-5 Diagnostic Sleep System (Philips Respironics, Murrysville, PA, USA). Recordings were manually scored by an experienced sleep technologist according to American Academy of Sleep Medicine criteria (version 3.0). Time spent in N1, N2, N3, and rapid eye movement (REM) sleep was calculated as a percentage of total sleep time.

Apnea was defined as a ≥90 per cent reduction in airflow signal amplitude lasting at least 10 s. Hypopnea was defined as a ≥30 per cent reduction in airflow lasting at least 10 s associated with either a ≥3 per cent oxygen desaturation or an arousal. The oxygen desaturation index (ODI) and stage-specific respiratory indices exported from the polysomnography software, including REM-AHI and non-rapid eye movement (NREM)-AHI, were retained for sensitivity analyses.

Hypoxemia duration was defined as total time spent with oxygen saturation <90 per cent during sleep (T90, min; SAV variable SURE90). The percentage of total sleep time spent below 90 per cent was used as an additional descriptive measure of hypoxemia burden.

### Neurodegenerative biomarker measurements

Venous blood samples were collected the morning after polysomnography following at least 8 h of fasting. Samples were centrifuged at 3000 rpm for 10 min to separate serum, and sera were stored at −80°C until biomarker analysis.

Seven serum biomarkers were measured: NSE, BDNF, CREB1, S100B, p-Tau/human pMAPT/pTAU, NfL, and GFAP. Biomarker concentrations were determined using biomarker-specific enzyme-linked immunosorbent assay kits (Elabscience; catalog numbers: NSE, E-EL-H1047; BDNF, E-EL-H0010; CREB1, E-EL-H0849; S100B, E-EL-H1297; p-Tau/human pMAPT/pTAU, E-EL-H5314; NfL, E-EL-H0741; and GFAP, E-EL-H6093).

According to the manufacturer’s accessible documentation, the E-EL-H5314 assay targets human pMAPT/pTAU (phosphorylated microtubule-associated protein tau), but no site-specific phospho-epitope such as p-tau181, p-tau217, or p-tau231 is explicitly provided. Therefore, we report this analyte conservatively as p-Tau/human pMAPT/pTAU rather than as a site-specific phospho-tau subtype. MAPT denotes microtubule-associated protein tau.

All assays were performed in the Harran University Physiology Research Laboratory under the manufacturer’s recommended conditions. A double-blind procedure was applied: laboratory personnel performing the assays and investigators conducting the statistical analyses were blinded to sample identity.

### Statistical analysis

Statistical analyses were performed using IBM SPSS Statistics for Windows, version 25.0 (IBM Corp., Armonk, NY, USA). Continuous variables are presented as mean ± standard deviation or median (interquartile range), as appropriate, and categorical variables are presented as *n* (%). Exact *p* values are reported throughout.

The analytic sequence was as follows. First, whole-group Spearman correlations were computed between respiratory burden measures (AHI, ODI, and T90) and each serum biomarker in the OSA cohort. Second, primary multivariable linear models evaluated continuous stage percentages and respiratory variables with adjustment for age and sex. NSE, S100B, BDNF, CREB1, and NfL were log-transformed because of right-skewed distributions.

Interaction models directly tested whether sleep-stage composition altered hypoxemia-biomarker relationships by fitting ODI × REM per cent models for BDNF and ODI × N3 per cent models for NfL, with age and sex covariates. Additional sensitivity analyses examined REM-AHI and NREM-AHI in age- and sex-adjusted models.

Median-split stage analyses were retained as exploratory sensitivity analyses using OSA-cohort medians for REM per cent, N1 per cent, N2 per cent, and N3 per cent. Stage-stratified subgroup correlations were considered exploratory; Bonferroni correction was applied within each stratum across 21 tests (7 biomarkers × 3 respiratory indices). Scatter plots, studentized residuals, Cook’s distance, and robust-regression sensitivity analyses were used to evaluate whether key findings were driven by influential observations. Available-case analysis was used for biomarker models, yielding assay-specific sample sizes from *n* = 70 to *n* = 71. Two-sided *p* < .05 was considered statistically significant.

## Results

### Cohort characteristics

A total of 91 adults underwent overnight polysomnography and morning blood sampling. Of these, 82 met the diagnostic criterion for OSA (AHI ≥5 events/h) and comprised the primary analytic cohort. Mean age was 50.4 ± 12.2 years, and 52 participants (63.4 per cent) were male. Biomarker availability ranged from 70 to 71 participants depending on assay availability. Baseline demographic, comorbidity, and sleep-study characteristics are summarized in Table 1.

**Table 1:** Baseline characteristics of the primary OSA cohort (AHI ≥5 events/h; *n* = 82)

Characteristic	Value
Age, y	50.4 ± 12.2
Male sex, *n* (%)	52 (63.4)
BMI, kg/m^2^	33.0 ± 5.9
Hypertension, *n* (%)	30 (36.6)
Diabetes mellitus, *n* (%)	22 (26.8)
Coronary artery disease, *n* (%)	19 (23.2)
Hypercholesterolemia, *n* (%)	44 (53.7)
Prior cerebrovascular disease, *n* (%)	5 (6.1)
AHI, events/h	27.8 (13.1–59.9)
ODI, events/h	25.9 (11.4–55.8)
T90, min	14.1 (1.7–83.8)
%TST with SpO2 <90%	3.2 (0.4–15.8)
Minimum SpO2, %	75.0 (63.0–85.0)
Total sleep time, min	374.1 ± 70.8
Sleep efficiency, %	83.2 ± 12.7
REM sleep, %TST	9.6 ± 6.2
N1 sleep, %TST	21.0 ± 14.2
N2 sleep, %TST	62.4 ± 12.6
N3 sleep, %TST	7.0 ± 6.8

Data are presented as mean ± SD, median (interquartile range), or *n* (%), as appropriate. T90 indicates total time spent with oxygen saturation <90 per cent during sleep.

### Whole-group correlations between respiratory burden and biomarkers

Across the OSA cohort, BDNF showed inverse correlations with ODI (rho = −0.299, *p* = .011) and AHI (rho = −0.277, *p* = .020). No other whole-group correlations between respiratory indices and biomarkers reached conventional statistical significance (Table 2).

**Table 2:** Whole-group Spearman correlations between respiratory indices and serum biomarkers in OSA

Biomarker	*n*	AHI rho	AHI *P*	ODI rho	ODI *P*	T90 rho	T90 *P*
NSE	71	−0.031	.799	−0.002	.985	−0.047	.699
GFAP	71	0.097	.420	0.097	.420	0.184	.125
p-Tau/human pMAPT/pTAU	71	0.166	.166	0.191	.111	0.163	.174
S100B	70	0.094	.440	0.125	.303	0.127	.296
BDNF	71	−0.277	.020	−0.299	.011	−0.148	.217
CREB1	71	−0.070	.561	−0.033	.786	−0.008	.945
NfL	71	0.071	.554	0.181	.131	0.120	.321

Exact *p* values are shown. T90 indicates total time spent with oxygen saturation <90 per cent during sleep.

### Age- and sex-adjusted models

**Figure 1:**
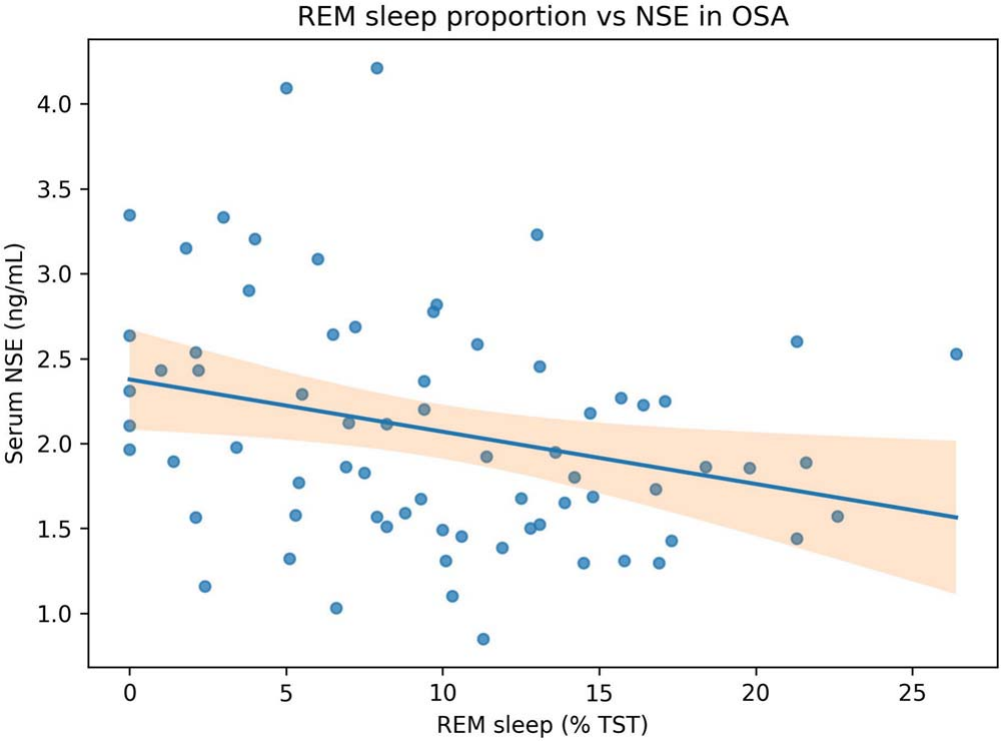
Relationship between REM sleep percentage and serum NSE in the OSA cohort. Points represent individual participants. The fitted line shows an inverse relationship, consistent with higher NSE at lower REM percentage.

**Figure 2:**
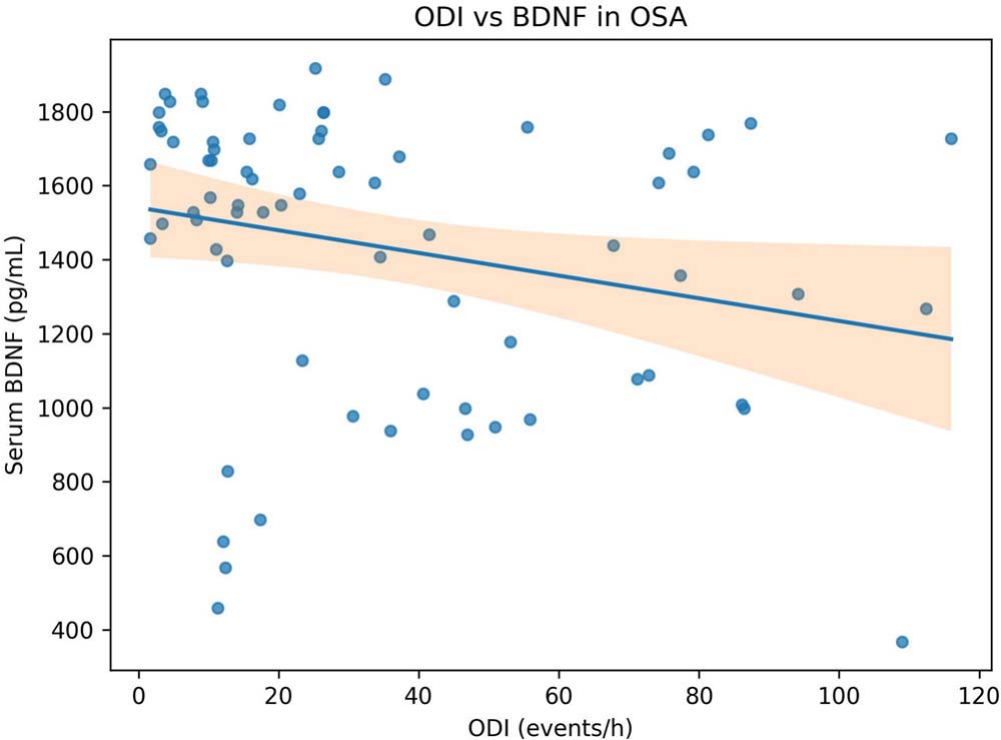
Relationship between ODI and serum BDNF in the overall OSA cohort. Points represent individual participants. The fitted line shows an inverse relationship.

In the primary adjusted analyses, lower REM percentage was associated with higher ln(NSE) (beta = −0.0138 per 1 per cent REM; 95% CI = −0.026 to −0.001; *p* = .033). By contrast, continuous N1 percentage was not significantly related to ln(S100B) (beta = 0.0061; *p* = .223), and continuous N2 percentage was not significantly related to p-Tau/human pMAPT/pTAU (beta = 0.9726; *p* = .509) (Table 3). Representative scatter plots are shown in Figures 1 and 2.

**Table 3:** Primary age- and sex-adjusted linear models and stage-specific sensitivity analyses

Outcome	Predictor term	*n*	beta	95% CI	*P* value
ln(NSE)	REM percentage, per 1%	71	−0.0138	−0.026 to −0.001	.033
ln(S100B)	N1 percentage, per 1%	70	0.0061	−0.004 to 0.016	.223
p-Tau/human pMAPT/pTAU	N2 percentage, per 1%	71	0.9726	−1.950 to 3.895	.509
ln(BDNF)	ODI x REM percentage	71	0.0004	−0.000 to 0.001	.079
ln(NfL)	ODI x N3 percentage	71	−0.0005	−0.001 to 0.000	.118
p-Tau/human pMAPT/pTAU	REM-AHI, per 1 event/h	71	1.799	0.534 to 3.063	.006
GFAP	REM-AHI, per 1 event/h	71	2.094	0.281 to 3.907	.024

All models were adjusted for age and sex. Interaction models also included the corresponding main effects. Log-transformed outcomes are indicated explicitly in the Outcome column.

### Direct interaction tests and robustness checks

The ODI × REM per cent interaction term for ln(BDNF) was directionally consistent with stronger inverse ODI-BDNF coupling at lower REM per cent, but it did not reach conventional significance (beta = 0.000412; 95% CI = −0.000049 to 0.000872; *p* = .079). Similarly, the ODI × N3 per cent interaction term for ln(NfL) was directionally similar but non-significant (beta = −0.000526; 95% CI = −0.001190 to 0.000138; *p* = .118) (Table 3).

Visual inspection of scatter plots, studentized residuals, Cook’s distance, and robust-regression sensitivity analyses suggested that the REM per cent-NSE finding was stable, whereas the interaction terms remained directionally similar but were attenuated.

### Exploratory median-split analyses

Using OSA-cohort medians (REM 9.4 per cent, N1 18.9 per cent, N2 63.65 per cent, and N3 5.4 per cent of total sleep time), short REM was associated with higher NSE (2.29 ± 0.78 vs 1.88 ± 0.54 ng/mL; *p* = .013), and long N1 was associated with higher S100B (323.37 ± 159.02 vs 242.15 ± 124.18 pg/mL; *p* = .021). The previously emphasized N2-p-Tau comparison was not significant in the final available-case reanalysis (1174.03 ± 161.46 vs 1130.94 ± 176.17 pg/mL; *p* = .247) (Table 4).

**Table 4:** Exploratory median-split analyses for key stage contrasts

Comparison	Biomarker	*n* (short/long)	Group values	*P* value
Short REM vs long REM	NSE, ng/mL	34/37	2.29 ± 0.78 vs 1.88 ± 0.54	.013
Short N1 vs long N1	S100B, pg/mL	36/34	242.15 ± 124.18 vs 323.37 ± 159.02	.021
Short N2 vs long N2	p-Tau/human pMAPT/pTAU, pg/mL	36/35	1130.94 ± 176.17 vs 1174.03 ± 161.46	.247

Group values are mean ± SD. Stage cut points were the OSA-cohort medians: REM 9.4 per cent, N1 18.9 per cent, N2 63.65 per cent, and N3 5.4 per cent of total sleep time.

### Exploratory stage-stratified correlations

Among raw stage-stratified correlations, the strongest finding was an inverse short-REM AHI-BDNF correlation (rho = −0.550, raw *p* = .00075) accompanied by an inverse short-REM ODI-BDNF correlation (rho = −0.500, raw *p* = .0026). Additional raw-significant findings were observed for BDNF and GFAP in long N1 and short N2 strata and for S100B with T90 in the short-REM stratum. After Bonferroni correction within each stratum, however, only the short-REM AHI-BDNF correlation remained significant (adjusted *p* = .0158) (Table 5).

**Table 5:** Raw-significant stage-stratified correlations and Bonferroni-adjusted *p* values

Stratum	Biomarker	Respiratory index	*n*	rho	Raw *P*	Adjusted *P*
Long N1	GFAP	T90	34	0.384	.025	.528
Long N1	BDNF	AHI	34	−0.356	.039	.815
Long N1	BDNF	ODI	34	−0.346	.045	.944
Short N2	GFAP	T90	36	0.378	.023	.486
Short N2	BDNF	AHI	36	−0.364	.029	.610
Short N2	BDNF	ODI	36	−0.345	.039	.828
**Short REM**	**BDNF**	**AHI**	**34**	**−0.550**	**<.001**	**.016**
Short REM	BDNF	ODI	34	−0.500	.003	.055
Short REM	S100B	T90	33	0.393	.024	.501

Bonferroni correction was applied within each stratum across 21 tests (7 biomarkers × 3 respiratory indices). Only the short-REM AHI-BDNF correlation remained significant after adjustment.

### Stage-specific respiratory index sensitivity analyses

**Figure 3:**
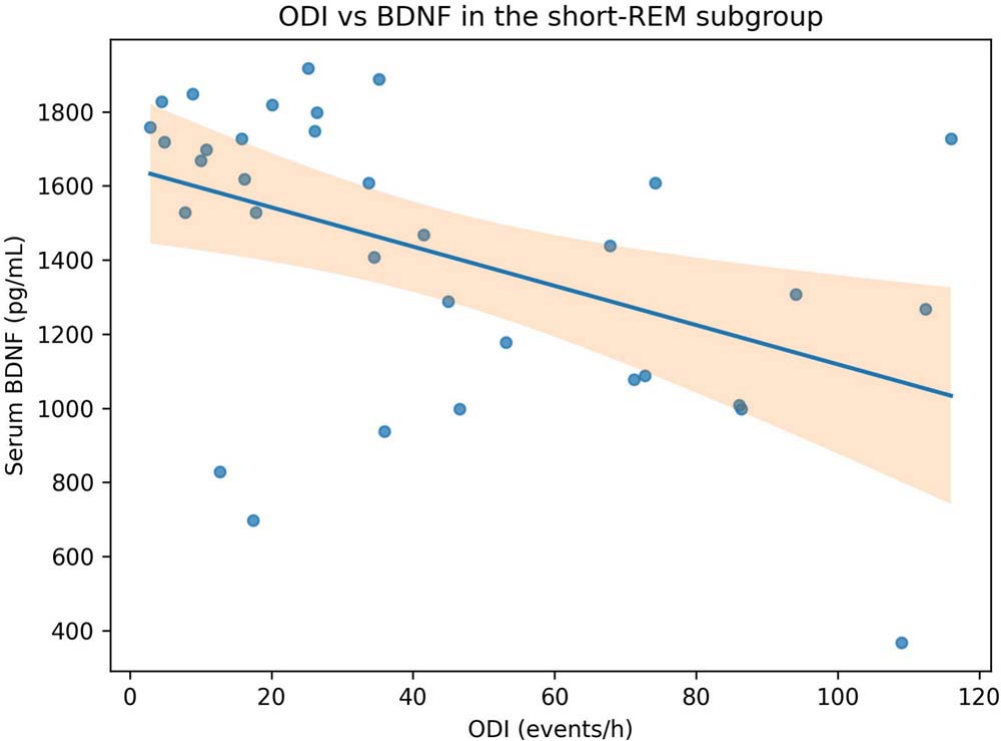
Relationship between ODI and serum BDNF in the short-REM subgroup. Points represent individual participants. The fitted line shows a stronger inverse trend than in the overall cohort.

**Figure 4:**
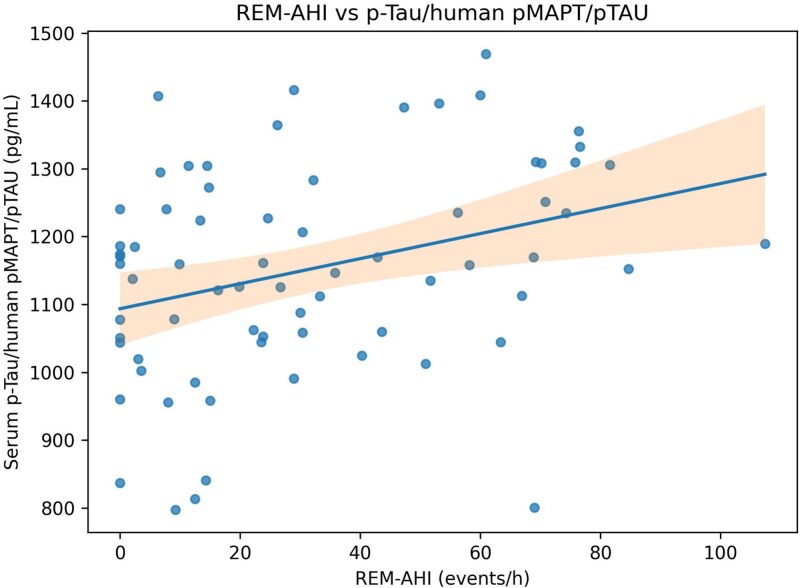
Relationship between REM-specific AHI and serum p-Tau/human pMAPT/pTAU. Points represent individual participants. The fitted line shows a positive relationship.

In age- and sex-adjusted sensitivity models, REM-AHI was positively related to p-Tau/human pMAPT/pTAU (beta = 1.799; 95% CI = 0.534 to 3.063; *p* = .006) and GFAP (beta = 2.094; 95% CI = 0.281 to 3.907; *p* = .024). NREM-AHI was not significantly related to these biomarkers. Severity-based interaction analyses using AHI ≥15 and AHI ≥30 thresholds did not identify significant effect modification for key BDNF, NfL, or S100B models (all interaction *p* > .05). Figures 3 and 4 illustrate selected exploratory sensitivity relationships.

## Discussion

In this cross-sectional OSA cohort, lower REM sleep showed the most consistent relationship with an adverse peripheral biomarker profile, specifically higher serum NSE, while higher respiratory burden in the cohort as a whole was associated with lower BDNF. By contrast, evidence that sleep-stage composition formally altered hypoxemia-biomarker coupling was attenuated after continuous age- and sex-adjusted modeling, direct interaction testing, multiplicity correction, and robustness analyses. Overall, the data support sleep architecture as a source of biomarker heterogeneity in OSA, but they provide only exploratory evidence for stage-dependent effect modification.

### REM sleep and NSE

NSE is a marker of neuronal soma injury, and the inverse relationship between REM percentage and NSE was the most stable signal across the analytic hierarchy of this study, persisting in both continuous adjusted models and exploratory median-split analyses. This observation is concordant with prior evidence that serum NSE can be elevated in OSA and supports the possibility that reduced REM sleep identifies a subgroup with greater neuronal vulnerability [10].

REM sleep is strongly implicated in synaptic plasticity and memory consolidation. A reduction in REM may therefore represent a vulnerable state in which intermittent hypoxia and sleep fragmentation are accompanied by greater neuronal stress. The persistence of the REM-NSE result in robust-regression sensitivity analyses supports a cautious but consistent interpretation.

### BDNF, hypoxemia, and exploratory stage dependence

Whole-group inverse correlations of BDNF with ODI and AHI support the possibility that greater respiratory burden is accompanied by diminished neurotrophic support in at least a subset of patients with OSA. Prior experimental and clinical work has suggested that BDNF may act as a compensatory neuroprotective signal in OSA, but published circulating results remain heterogeneous [3, 13–15].

Our direct ODI × REM per cent interaction model for BDNF was directionally consistent with stronger adverse ODI-BDNF coupling at lower REM per cent, and the short-REM AHI-BDNF correlation remained significant after multiplicity correction. However, because the formal interaction term did not reach conventional significance, these data are better viewed as hypothesis-generating evidence that reduced REM sleep may identify a more biologically vulnerable phenotype rather than proof of effect modification.

### Light sleep, astroglial stress, and stage-specific respiratory burden

The exploratory long-N1 and higher-S100B finding is consistent with the concept that expanded light sleep and sleep fragmentation may accompany astroglial stress or blood–brain barrier disturbance [8–10]. Nevertheless, the corresponding continuous N1 per cent model was not significant after age and sex adjustment, so this signal should be considered secondary.

REM-specific respiratory burden provided additional exploratory information. REM-AHI was positively related to p-Tau/human pMAPT/pTAU and GFAP, whereas NREM-AHI was not. Although stage-specific hypoxemia-duration variables were not available in the exported dataset, these findings raise the possibility that respiratory events concentrated during REM sleep may be biologically relevant for tau-related and astroglial injury pathways [8, 11, 12].

### Deep sleep and neurobiological resilience

The ODI × N3 per cent interaction for NfL was directionally consistent with weaker adverse hypoxemia-NfL coupling when deep sleep was preserved, but the result did not reach statistical significance. This directional pattern remains biologically plausible because slow-wave sleep has been linked to metabolite clearance and because chronic sleep disruption can accelerate tau-related neurodegeneration in experimental models [20, 21]. Even so, our data do not justify a strong claim that N3 formally modifies respiratory-biomarker relationships.

### CREB1 and GFAP null findings

Serum CREB1 did not show consistent relationships with sleep architecture or respiratory burden. This may reflect biological compartmentalization: the most relevant CREB processes in sleep–wake regulation and memory are intracellular and phosphorylation-dependent, which may not be well captured by peripheral CREB1 concentrations [19].

GFAP likewise did not demonstrate clear whole-group associations with OSA severity. Astrocytic cytoskeletal remodeling may be more closely linked to chronic neuroinflammation or more advanced neurodegeneration than to the cross-sectional biological stress captured in this cohort [8].

### Clinical implications

OSA is potentially modifiable, and a biomarker-informed framework may help identify patients at greater risk of neurocognitive sequelae. Our data suggest that sleep architecture, particularly REM sleep, should be considered alongside conventional OSA severity indices when interpreting peripheral biomarkers. Larger longitudinal studies are needed to determine whether REM-linked biomarker patterns track cognitive trajectories, respond to therapy, and generalize across independent cohorts [4, 15, 22, 23].

### Limitations and future directions

Several limitations warrant consideration. First, the cross-sectional design precludes causal inference. Second, biomarker availability varied across assays, so analyses used available-case data rather than a complete-case biomarker panel. Third, the exploratory median-split approach can reduce power and obscure dose–response relationships, which is why continuous adjusted models were prioritized.

Fourth, biomarker measurements were obtained from peripheral blood and may be influenced by systemic inflammation, comorbidities, or blood–brain barrier dynamics. Fifth, neurocognitive testing and neuroimaging were not available, limiting direct linkage between serum biomarkers and clinical outcomes. Sixth, stage-specific hypoxemia-duration variables were not available in the exported polysomnography dataset. Finally, the commercial ELISA assay used for p-Tau identifies the analyte broadly as human pMAPT/pTAU in the manufacturer’s accessible documentation, without explicit definition of the phospho-tau epitope site. Accordingly, our results should be interpreted as reflecting a general phosphorylated tau signal rather than a site-specific phospho-tau species such as p-tau181, p-tau217, or p-tau231.

## Conclusion

Reduced REM sleep showed the clearest and most reproducible association with an adverse peripheral biomarker profile in adults with OSA, particularly higher serum NSE. Sleep architecture may contribute to biomarker heterogeneity beyond conventional OSA severity indices; however, evidence for stage-dependent modification of hypoxemia-biomarker relationships remains exploratory. Larger longitudinal studies incorporating cognitive outcomes and treatment-response data are needed to confirm the clinical relevance of these findings.

## Supplementary Material

07_Graphical_Abstract_Optional_zpag046

## Data Availability

The data underlying this article are not publicly available because of ethical and privacy restrictions. De-identified participant data may be made available from the corresponding author upon reasonable request and with approval from the relevant institutional ethics committee.

## Notes

Institution where the work was performed: Harran University Sleep Clinic, Sanliurfa, Turkiye

## References

[1] Yang Q, Wang Y, Feng J, Cao J, Chen B. Intermittent hypoxia from obstructive sleep apnea may cause neuronal impairment and dysfunction in central nervous system: the potential roles played by microglia. *Neuropsychiatr Dis Treat*. 2013;9:1077–1086. 10.2147/NDT.S49868 23950649 PMC3742344

[2] Qi KR, Chen X, Si JC, Yang SC. Research progress on chronic intermittent hypoxia and cognitive impairment [in Chinese]. *Sheng Li Xue Bao*. 2024;76(5):752–760 39468811

[3] Xie H, Leung KL, Chen L, et al. Brain-derived neurotrophic factor rescues and prevents chronic intermittent hypoxia-induced impairment of hippocampal long-term synaptic plasticity. *Neurobiol Dis*. 2010;40(1):155–162. 10.1016/j.nbd.2010.05.020 20553872

[4] Yaffe K, Laffan AM, Harrison SL, et al. Sleep-disordered breathing, hypoxia, and risk of mild cognitive impairment and dementia in older women. *JAMA.* 2011;306(6):613–619. 10.1001/jama.2011.1115 21828324 PMC3600944

[5] Jaromirska J, Kaczmarski P, Strzelecki D, Sochal M, Białasiewicz P, Gabryelska A. Shedding light on neurofilament involvement in cognitive decline in obstructive sleep apnea and its possible role as a biomarker. *Front Psych*. 2023;14:1289367. 10.3389/fpsyt.2023.1289367 PMC10720906 38098628

[6] Kahn OI, Dominguez SL, Glock C, et al. Secreted neurofilament light chain after neuronal damage induces myeloid cell activation and neuroinflammation. *Cell Rep*. 2025;44(3):115382. 10.1016/j.celrep.2025.115382 40056413

[7] Arslan B, Semsi R, Iriz A, Sepici DA. The evaluation of serum brain-derived neurotrophic factor and neurofilament light chain levels in patients with obstructive sleep apnea syndrome. *Laryngoscope Investig Otolaryngol*. 2021;6(6):1466–1473. 10.1002/lio2.683 PMC8665460 34938889

[8] Sofroniew MV, Vinters HV. Astrocytes: biology and pathology. *Acta Neuropathol*. 2010;119(1):7–35. 10.1007/s00401-009-0619-8 20012068 PMC2799634

[9] Sorci G, Bianchi R, Riuzzi F, Arcuri C, Giambanco I, Donato R. S100B protein, a damage-associated molecular pattern protein in the brain and heart, and beyond. *Cardiovasc Psychiatry Neurol*. 2010;2010:656481. 10.1155/2010/656481 20827421 PMC2933911

[10] Rezaei F, Abbasi H, Sadeghi M, Imani MM. The effect of obstructive sleep apnea syndrome on serum S100B and NSE levels: a systematic review and meta-analysis of observational studies. *BMC Pulm Med*. 2020;20(1):31. 10.1186/s12890-020-1063-8 32024492 PMC7003338

[11] Huang ZW, Zeng HX, Huang YP, et al. The relationship between obstructive sleep apnea and circulating tau levels: a meta-analysis. *Brain Behav*. 2023;13(4):e2972. 10.1002/brb3.2972 36938834 PMC10097049

[12] Chen YS, Chen MH, Wang PM, Lu CH, Chen HL, Lin WC. Increased levels of plasma Alzheimer’s disease biomarkers and their associations with brain structural changes and carotid intima-media thickness in cognitively normal obstructive sleep apnea patients. *Diagnostics (Basel)*. 2022;12(7):1522. 10.3390/diagnostics12071522 35885428 PMC9324500

[13] Gabryelska A, Turkiewicz S, Ditmer M, Sochal M. Neurotrophins in the neuropathophysiology, course, and complications of obstructive sleep apnea-a narrative review. *Int J Mol Sci*. 2023;24(3):1808. 10.3390/ijms24031808 36768132 PMC9916304

[14] Flores KR, Viccaro F, Aquilini M, et al. Protective role of brain-derived neurotrophic factor (BDNF) in obstructive sleep apnea syndrome (OSAS) patients. *PloS One*. 2020;15(1):e0227834. 10.1371/journal.pone.0227834 31951637 PMC6968866

[15] Karwowska U, Kudrycka A, Pierzchała K, et al. Twelve-month CPAP therapy modulates BDNF levels in patients with severe obstructive sleep apnea: implications for metabolic and treatment compliance. *Int J Mol Sci*. 2025;26(12):5855. 10.3390/ijms26125855 40565316 PMC12192732

[16] Wang WH, He GP, Xiao XP, Gu C, Chen HY. Relationship between brain-derived neurotrophic factor and cognitive function of obstructive sleep apnea/hypopnea syndrome patients. *Asian Pac J Trop Med*. 2012;5(11):906–910. 10.1016/S1995-7645(12)60169-2 23146807

[17] Deuschle M, Schredl M, Wisch C, et al. Serum brain-derived neurotrophic factor (BDNF) in sleep-disordered patients: relation to sleep stage N3 and rapid eye movement (REM) sleep across diagnostic entities. *J Sleep Res*. 2018;27(1):73–77. 10.1111/jsr.12577 28656632

[18] Coluk Y, Yildirim G, Yildirmak S, Gulec Peker EG. Altered brain-derived neurotrophic factor levels and oxidative stress in REM sleep deprivation: a rat model study. *BMC Neurol*. 2025;25(1):122. 10.1186/s12883-025-04127-2 40119302 PMC11927282

[19] Koch JM, Stengel M, Aldenhoff JB. CREB-phosphorylation is increased via the 5-HT1A receptor: reason for the modulatory effects of SSRI on sleep/wake regulation? *Exp Clin Endocrinol Diabetes*. 2003;111(06):P54. 10.1055/s-2003-817596

[20] Xie L, Kang H, Xu Q, et al. Sleep drives metabolite clearance from the adult brain. *Science.* 2013;342(6156):373–377. 10.1126/science.1241224 24136970 PMC3880190

[21] Martin SC, Joyce KK, Lord JS, et al. Sleep disruption precedes forebrain synaptic tau burden and contributes to cognitive decline in a sex-dependent manner in the P301S tau transgenic mouse model. *eNeuro*. 2024;11(6):ENEURO.0004-24.2024. 10.1523/ENEURO.0004-24.2024 PMC11209651 38858068

[22] Leng Y, McEvoy CT, Allen IE, Yaffe K. Association of sleep-disordered breathing with cognitive function and risk of cognitive impairment: a systematic review and meta-analysis. *JAMA Neurol*. 2017;74(10):1237–1245. 10.1001/jamaneurol.2017.2180 28846764 PMC5710301

[23] Kylstra WA, Aaronson JA, Hofman WF, Schmand BA. Neuropsychological functioning after CPAP treatment in obstructive sleep apnea: a meta-analysis. *Sleep Med Rev*. 2013;17(5):341–347. 10.1016/j.smrv.2012.09.002 23063416

