# Supplementary material for: Sleep architecture and serum biomarker heterogeneity in obstructive sleep apnea: a cross-sectional study
Source: Sleep Adv. 2026 May 22;7(2):zpag046. doi: 10.1093/sleepadvances/zpag046 (PMC13221959; doi:10.1093/sleepadvances/zpag046)
Supplement: 07_Graphical_Abstract_Optional_zpag046 [file 07_graphical_abstract_optional_zpag046.pdf]

Graphical Abstract

SLEEPADV-2026-014 | Final SAV-based revision

Study design

- OSA cohort: AHI >= 5 events/h
- Primary cohort n = 82
- Biomarker availability: n = 70-71
- Age/sex-adjusted linear models
- Median-split analyses retained as exploratory

Most reproducible finding

- Lower REM% -> higher serum NSE
- Adjusted model: beta = -0.0138
- p = 0.033
- Short-REM subgroup also showed higher NSE
- Signal remained stable in robustness checks

Hypoxemia-related findings

- Whole group: higher ODI/AHI -> lower BDNF
- REM% x ODI and N3% x ODI interactions
- Directionally consistent with hypothesis
- Did not reach conventional significance
- Interpret as exploratory

Conclusion: Sleep architecture may contribute to biomarker heterogeneity in OSA, but stage-dependent modification of hypoxemia-biomarker coupling should be interpreted cautiously.
